# Air Pollution Monitoring Using Cost-Effective Devices Enhanced by Machine Learning

**DOI:** 10.3390/s25051423

**Published:** 2025-02-26

**Authors:** Yanis Colléaux, Cédric Willaume, Bijan Mohandes, Jean-Christophe Nebel, Farzana Rahman

**Affiliations:** 1 National School for Statistics and Data Analysis (ENSAI), Blaise Pascal BP37203, 35172 Bruz, France; yanis.colleaux@eleve.ensai.fr; 2 National Graduate School of Engineering of Caen (ENSICAEN), 6 Boulevard Maréchal Juin—CS 45053, 14050 Caen, France; cedric.willaume@ecole.ensicaen.fr; 3 Technocomm Consulting Ltd., The Barn, Highwood Farm, Long Lane, Newbury RG14 2TB, UK; bijan@technocommconsulting.com; 4 Faculty of Engineering, Computing and the Environment, Kingston University, Holmwood House, Grove Crescent, Kingston upon Thames KT1 2EE, UK; farzana@kingston.ac.uk

**Keywords:** air pollution monitoring, low-cost sensors, electrochemical sensors, non-dispersive infrared sensors, sensor performance variability, measurement correction, sensor calibration, data-driven correction, machine learning, multiple linear regression models

## Abstract

Given the significant impact of air pollution on global health, the continuous and precise monitoring of air quality in all populated environments is crucial. Unfortunately, even in the most developed economies, current air quality monitoring networks are largely inadequate. The high cost of monitoring stations has been identified as a key barrier to widespread coverage, making cost-effective air quality monitoring devices a potential game changer. However, the accuracy of the measurements obtained from low-cost sensors is affected by many factors, including gas cross-sensitivity, environmental conditions, and production inconsistencies. Fortunately, machine learning models can capture complex interdependent relationships in sensor responses and thus can enhance their readings and sensor accuracy. After gathering measurements from cost-effective air pollution monitoring devices placed alongside a reference station, the data were used to train such models. Assessments of their performance showed that models tailored to individual sensor units greatly improved measurement accuracy, boosting their correlation with reference-grade instruments by up to 10%. Nonetheless, this research also revealed that inconsistencies in the performance of similar sensor units can prevent the creation of a unified correction model for a given sensor type.

## 1. Introduction

Air pollution has significant implications for public health, climate change, and urban planning [1,2,3,4]. According to the World Health Organization, air pollution is responsible for nearly 7 million premature deaths yearly and is the second highest risk factor for noncommunicable diseases [5,6,7,8,9,10,11]. To tackle this critical global issue, it is essential to implement effective monitoring strategies and eventually take action to mitigate pollution [2,5,12,13,14,15,16]. Although the first air quality monitoring stations were established in the 1950s [17], and nowadays most major cities have such systems, the financial burden and limited accessibility of suitable instrumentation have posed significant barriers to these efforts [4,18]. The high cost of monitoring systems is attributed to the need to invest in expensive reference-grade instruments to ensure accurate measurements [19,20]. In addition, these instruments have suffered from limited spatial coverage due to their lack of portability, in addition to placement and availability issues [21].

In response to these challenges, cost-effective air quality monitoring devices have been developed using low-cost sensors [22]. The low-cost sensors are not only significantly cheaper than traditional stations but also typically occupy less space and can be mobile [23]. In recent years, affordable devices have emerged as viable alternatives, facilitating wider deployment and frequently enabling near-real-time data collection. Nonetheless, these sensors still face challenges related to calibration, accuracy, reliability, and standardisation [21,24].

Although improving the hardware of affordable sensors is challenging without significantly increasing costs or compromising practicality, leveraging software to enhance their performance is a highly promising approach [25]. For instance, by comprehending how external factors affect sensor measurements, it becomes feasible to apply corrections that yield more reliable values [26,27]. Ultimately, the goal is to improve the observation quality of these devices to a level where they can perform tasks that are currently exclusive to traditional monitoring stations [28].

The objective of this study is to assess whether machine learning algorithms implemented on a microcontroller can improve readings from a set of low-cost sensors designed to measure CO, O_3_, and CO_2_ concentrations. The key research contributions are as follows:

- Quantitative data on gas cross-sensitivities and the influence of environmental factors on readings from low-cost sensors, i.e., electrochemical and non-dispersive infrared sensors.
- Quantitative evidence of significant inter-unit inconsistency among low-cost gas sensors, especially among electrochemical sensors.
- Demonstration of significant performance improvement achieved by low complexity machine learning models, i.e., Multiple Linear Regression models, that offer some explainability.

This manuscript begins by reviewing the benefits of cost-effective air pollution monitoring devices, their current technical limitations, and methods to improve their measurement accuracy. It then details the sensors under investigation and the data collection process. Following this, a comprehensive description of the selected methodology to enhance measurement accuracy is given. Subsequently, experimental results are presented. Finally, the manuscript concludes with discussions and a summary of key conclusions.

## 2. Related Work

The development and availability of cost-effective air pollution monitoring devices have instigated a significant body of research dedicated to assessing their suitability for the intended applications. While the literature underscores their remarkable potential, it also highlights their inherent technical limitations. Fortunately, advancements in machine learning have enabled the development of more intelligent sensors, capable of accounting for factors such as sensor drift and cross-sensitivity, thereby delivering more accurate corrected readings.

### 2.1. Advantages of Cost-Effective Air Pollution Monitoring Devices

In large population centres, such as Shanghai, Hong Kong, Delhi, Dhaka, Dallas, and London, that suffer from severe air pollution challenges [29,30] often exacerbated by rapid urbanisation, industrial activities, and vehicular emissions [31], the usage of traditional air quality monitoring stations is indispensable yet insufficient on their own [32,33]. As of today, London has only 83 air quality monitoring stations, fewer than one station per 100,000 inhabitants, which is clearly insufficient given that air quality can vary significantly between neighbouring streets [34]. Coverage is even lower in countries that are the most affected by air pollution, e.g., there are only 11 stations in Bangladesh. Indeed, a main obstacle to station deployment is their cost, which is typically over USD 10,000 per unit without considering installation and maintenance charges. As low-cost sensors have shown promise in addressing the increasingly pressing challenge of urban air pollution [35], their deployment, either in tandem with or independently from existing networks [36], can achieve better spatial coverage, improved data granularity, and eventually deliver more timely interventions [4,30].

The primary appeal of these cost-effective devices lies in their scalability and the flexibility of their deployment. Indeed, their competitive cost allows for far more extensive deployment than would be feasible with expensive reference-grade monitors [25]. In sprawling metropolises, the availability of only a limited number of official monitoring stations cannot adequately capture localised pollution hotspots or micro-environments [32], resulting in data blind spots. Low-cost sensors, on the other hand, can be deployed in more significant numbers within residential districts, near industrial zones, and along congested traffic corridors [37]. This expanded network translates into a more precise understanding of how pollution varies across neighbourhoods, enabling policymakers to tailor interventions to the most affected areas [30].

Second, the portability and relatively small form factor of these devices make them far easier to install and maintain. While larger, reference-grade stations may require substantial ground space, stable power sources [4], and climate control units, the sensors can be placed on rooftops, street lights, or moving vehicles [25]. This ease of placement is especially valuable in densely packed cities such as Hong Kong and London, where real estate is at a premium and infrastructural constraints are significant. Moreover, while traditional stations often require regular visits from specialised personnel, these sensors can often be serviced, calibrated, and updated remotely, reducing operational costs and minimising downtime [38].

Third, as cost-effective air pollution monitoring devices are usually integrated with an Internet of Things (IoT) platform, real-time or near-real-time data streams from a dense network can be invaluable for immediate decision making. In cities prone to episodic pollution events, such as the winter smog in Delhi or the dust storms that occasionally affect Dallas, these data allow local authorities to promptly issue health advisories [31], divert traffic flows, or temporarily restrict polluting activities [28]. The ability to respond proactively can mitigate the severity of pollution events and reduce residents’ exposure to harmful particulate matter and gases [30,39].

Fourth, the affordability and user-friendliness of these devices may encourage citizen engagement [4] and foster collaborative monitoring, particularly in regions where public awareness is crucial for policy support. Initiatives such as Breathe London [40] and a community-led effort in Hong Kong [41] demonstrate how crowd-sourced air quality data can help individuals manage air pollution exposure and related health risk at an urban scale. The large-scale deployment of low-cost sensors also allows for the tracking of local pollution patterns in real time [25] and holding industries or municipal bodies accountable when levels exceed safe thresholds [42]. This democratisation of data creates a virtuous cycle of engagement [43], awareness, and policy responsiveness, thereby accelerating improvements in air quality management [30].

Finally, cost-effective air pollution monitoring aligns with several United Nations Sustainable Development Goals [31]. As large population centres face increasing pressure from rapid urbanisation, climate change, and public health crises, these sensors support cities in meeting international guidelines, reducing environmental inequalities, and safeguarding public health. Their ability to integrate seamlessly with digital platforms means local authorities, researchers, and citizens alike could access actionable insights, plan more sustainable infrastructure, and take steps to reduce pollution at its source.

### 2.2. Current Technical Limitations

Even if they have numerous advantages, cost-effective air pollution monitoring devices have limitations when compared against their reference-grade counterparts. Specifically, they depend on the technology of their individual sensors that can vary dramatically according to their target pollutants.

Whereas particulate matter is usually quantified by optical particle counters (OPCs) that focus a beam of light within the sensor and record its scattering using a photodetector, low-cost gas-sensing technologies can be classified into four main types [44]:

- Electrochemical (EC) sensors measure the current produced by electrochemical reactions with the target gas [45].
- Non-dispersive infrared (NDIR) sensors track reductions in infrared radiation when the gas passes through an active filter [46].
- Metal oxide semiconductor (MOS) sensors rely on gas–solid interactions that induce an electronic charge on the metal oxide surface.
- Photo-ionisation detection (PID) sensors employ ultraviolet (UV) light to ionise target molecules, convert the resulting ions into digital readings, and thus quantify the chemical content [47].

Despite the variety of these technologies, these sensors experience inaccuracies caused by the following factors: sensor drift and ageing, cross-sensitivity and interferences, manufacturing inconsistencies and a lack of standards, environmental factors, and limited dynamic range and saturation effects.

Due to the low-cost nature of these sensors, they are all subject to batch-to-batch variations in component quality, such as slight chemical deviations in sensor electrodes, which can shift baselines, introduce offset misalignments, and cause inconsistencies in sensitivity from unit to unit [19,38]. Furthermore, many sensors are shipped with minimal or no factory calibration, leaving users to rely on in-field calibration that may be inconsistent or inadequate. Even when calibration is provided, manufacturers may use unspecified and/or proprietary algorithms [4]. Moreover, when they provide correction equations, they are based on tests conducted in laboratories, where conditions are controlled and simplified, which often does not reflect real-world conditions and fails to account for complex interactions between different pollutants. Compounding this problem is the absence of unified testing guidelines or standardised protocols. Thus, one manufacturer’s ’validated’ data might not be comparable to another’s [21,48].

Another general characteristic of low-cost sensors is that their enclosure may lack adequate ingress protection, making them susceptible to water damage from rain or dew [49,50]. Furthermore, these enclosures often cannot endure prolonged exposure to UV light. In urban settings, high levels of electromagnetic interference can cause noise in the sensor’s electronics, especially if sufficient shielding is not provided. Furthermore, corrosion and oxidation can damage the metal components, negatively impacting the sensor’s electrical performance. Finally, low-cost sensors often produce raw signals rife with high-frequency variability, outliers, and artefacts brought on by electrical spikes or mechanical vibrations.

In addition to these general technical limitations, cost-effective air pollution monitoring devices suffer from a range of factors that are specific to their working mechanisms.

**Optical particle counters**

The main issue with optical particle counters is lens obstruction from dust or soot, skewing scattering intensity [51], and particulate accumulation in the sampling path compromising airflow, reducing the consistency of particle detection [52]. Consequently, regular calibration is needed, sometimes monthly or quarterly, which increases operational costs and complexity [53]. Additionally, condensation within the sampling chamber can distort scattering profiles and a sudden surge of particulates can clog the measurement chamber, leading to transient errors. Another issue is that particles of diverse sizes and compositions may scatter light differently, making it difficult to classify them accurately by size. Moreover, as aerosols can absorb moisture, artificially inflating particle diameter and varying refractive indices, high relative humidity can lead to overestimated concentrations [24].

**Electrochemical sensors**

Over time, electrochemical sensors experience degradation as their reactive electrolyte or electrode materials are consumed, altering their output. Whereas some sensors degrade within months, others maintain partial functionality for two or three years while accumulating biases [54]. Moreover, prolonged exposure to high or low temperatures and impurities can amplify drift by accelerating chemical reactions, depleting internal reagents or causing them to form condensation [55,56,57,58]. Finally, it has also been reported that ageing influences bias in voltage recordings at certain environmental ozone concentrations [59].

In addition to ageing, EC sensors often register cross-reactants, such as NO_2_ sensors also responding to O_3_, Cl_2_, and H_2_S, which inflates or distorts measurements [25,60,61]. High pollutant concentrations can also saturate electrochemical sensors, leaving the electrode’s reaction rate plateaued. Moreover, shifts in humidity and temperature can trigger spurious voltage changes or accelerate side reactions in the sensing chamber [62]. In particular, they are vulnerable to interference from water vapour, which, by occupying reactive sites on the metal oxide surface, leads to underestimations of pollutant levels [59,63,64,65].

**Non-dispersive infrared sensors**

Despite their growing popularity, non-dispersive infrared sensors face several inherent limitations that influence data fidelity, sensor lifespan, and overall utility in real-world deployments [66]. Depending on the quality of their filter design, they may be particularly vulnerable to interference from other infrared-absorbing gases. Such cross-sensitivity can inflate or depress readings based on the overlap in absorption spectra. Furthermore, condensation or particulate deposits on the detector degrade performance, diminishing the sensor’s responsiveness and reliable range [67,68]. Moreover, these sensors are prone to calibration drift, which is exacerbated by environmental factors such as temperature and humidity fluctuations [69,70].

While high-end NDIR systems employ sophisticated temperature-compensation circuits and superior optical filters, low-cost NDIR sensors lack such refinements, resulting in lower measurement accuracy [70]. Due to their limited temperature compensation capability, even minimal temperature changes can introduce sizeable measurement errors over time. Therefore, if recalibration is not performed regularly, readings can drift far from reality. Eventually, in the absence of a robust enclosure and/or advanced compensation algorithms, their data quality may be questionable in real-world deployments, i.e., where environmental conditions can vary widely [63,64].

**Photo-ionisation detection sensor**

Due to their fast response times and broad-spectrum sensitivity, photo-ionisation detection sensors are widely used, especially for detecting volatile organic compounds. However, selectivity is a major concern as they ionise all compounds with an ionisation potential below the lamp’s energy (typically 10.6 eV) [71]. This leads to interference from multiple volatile organic compounds, such as benzene, toluene, and xylene, which cannot be distinguished without additional separation techniques [47,72]. Additionally, sensor drift and stability degradation are key operational limitations, with reported drift values ranging between 2% and 15% per year [72]. Humidity and temperature variations also significantly affect PID performance, necessitating frequent recalibration [73]. Moreover, studies have shown that sensor accuracy degrades beyond acceptable error margins when exposed to relative humidity variations above 50% [57,71]. Furthermore, their limit of detection varies significantly from sub-ppb levels to above 2.5 ppm depending on the model and manufacturer [72]. Eventually, real-world performance validation has highlighted discrepancies between laboratory-calibrated PIDs and field-deployed versions, with studies indicating deviations of up to ±25% when compared with reference monitoring stations [57].

From an economic perspective, maintenance costs and power consumption present additional barriers to large-scale deployment. Indeed, they require periodic UV lamp replacement, increasing long-term operational costs beyond those of other LCS technologies such as MOS or EC sensors [72]. Additionally, power requirements are significantly higher due to the UV excitation mechanism, making them less suitable for battery-powered applications in mobile or remote monitoring systems.

**Metal oxide semiconductor sensors**

MOS sensors are known to deliver very high sensitivity but usually lack selectivity. Thus, they tend to group chemically similar volatile organic compounds into a single reading [74]. These sensors can also experience overload under heavy pollutant exposure, leading to ’burn-in’ that demands a lengthy recovery. Furthermore, MOS sensors may suffer from surface poisoning when contaminants are adsorbed onto the metal oxide, irreversibly changing its conductivity. In addition, both extreme temperatures and high humidity impact their performance [75,76]. Also, when the surfaces of these sensors become saturated, their ability to absorb pollutants is hindered [77,78]. Finally, hysteresis effects can arise as these sensors transition between high and low pollutant concentrations, impairing consistency in the readings [79].

### 2.3. Measurement Corrections

It is essential to address the aforementioned limitations to ensure that cost-effective devices can fulfil their intended functions in real-world usage conditions and achieve the desired impact on air pollution monitoring. Among different approaches compared, advanced machine learning techniques like support vector regression and deep-learning-based methods were found to deliver better results [48,80,81,82,83,84,85,86]. Recent advances in software calibration, sensor miniaturisation, and machine learning have significantly narrowed the performance gap between low-cost sensors and their high-end counterparts [25]. Using sophisticated algorithms [87], particularly those powered by artificial intelligence, their readings can be adjusted for temperature, humidity, and cross-sensitivity to other pollutants [27]. Moreover, the integration of additional data sources, such as meteorological variables, has been shown to enhance further sensor correction [25,26,28,88,89]. The continuous advancement in data analytics ensures that sensors become increasingly ’smarter’ over time, adapting to new conditions and providing reliable trend insights.

Although initial models explored for correcting low-cost sensor measurements relied on the simple, but interpretable, linear regression, more advanced models have generally been more reliable [85]. Among the many non-linear machine learning approaches, ensemble learning methods that use decision trees have proved particularly popular due to their ability to capture complex non-linear relationships in sensor responses, allowing them to deliver a strong performance in addressing cross-sensitivity and environmental interference. Among them, gradient boosting regression trees (GBRT) have been widely used to model non-linear relationships between sensor readings and meteorological factors. Compared to linear regression, GBRT was shown to improve the coefficient of determination (R^2^) from 0.36–0.51 to 0.68–0.76 for aerosol monitoring [88]. Another tree-based method, Random Forest (RF), has achieved significant popularity. Indeed, a recent study reported that RF-based corrections improved R^2^ values for both particulate matter with a diameter smaller than 10 µm (PM10) and 2.5 µm (PM2.5) and gaseous pollutants (SO_2_, NO_2_, CO, and O_3_) to a range of 0.70–0.99, with root mean squared error values between 4.05 and 17.79 µg/m^3^ for the gases [25]. Similarly, the usage of a multi-stage approach that incorporates RF, baseline drift correction, and empirical filters improved sensor accuracy to ±2.6 ppb for NO_2_, ±4.4 µg/m^3^ for PM10, and ±2.7 µg/m^3^ for PM2.5 [26]. Additionally, RF models have been found to perform well over extended time periods, showing stability for up to 16 weeks, whereas more traditional models deteriorate more rapidly [89].

Another category of machine learning models, i.e., artificial neural networks (ANNs), including deep learning models, has been extensively studied to enhance measurements due to their effectiveness in handling non-linearities in sensor data [62,89,90,91]. Whereas dynamic neural network models were shown to achieve significant error reductions (the mean absolute error) to less than 2 ppb for NO_2_, performance for O_3_ proved disappointing [89]. Similarly, ANN-based models proved efficient for certain gases, i.e., O_3_ and CO_2_, but not for others, i.e., NO_2_ [25]. More complex deep learning models, including hybrid ones, have also been designed to improve the precision of pollutant prediction. For example, a combination of convolutional neural networks with long- and short-term memory networks led the O_3_ accuracy to increase to 3.58% [25,86]. Despite the performance of these models, they are often much less interpretable than others, making them less desirable for regulatory applications in particular [50].

The absence of standard datasets, the diversity of sensor technologies and models, the regular introduction of new devices, the significant variability in real-life conditions, and the lack of standardised evaluation protocols and metrics render comparisons between machine learning approaches impractical. Moreover, the evaluation of a large range of models on the same dataset has revealed that the optimal method depends on the type of sensor and pollutant of interest. Indeed, besides RF and artificial neural networks, other models, such as generalised additive models and support vector regression, were also found to be optimal under certain conditions [88].

In conclusion, selecting an appropriate methodology must be tailored to the specific sensor, intended usage, potential needs for real-time and on-device processing, and explainability requirements.

## 3. Dataset: Co-Located Cost-Effective Device and Reference Station Measurements

### 3.1. Data Collection

This study evaluates the performance of cost-effective gas sensors and their enhancement using machine learning. Its focus is on measurements of carbon monoxide (CO), ozone (O_3_), and carbon dioxide (CO_2_), which are captured by two electrochemical sensors and one non-dispersive infrared sensor, respectively. EC sensors were selected as they proved to be the only small-size low-cost sensors available to measure O_3_ and CO. Indeed, alternative measurement techniques would have necessitated significantly more expensive equipment, which must be maintained at a constant temperature. Regarding CO_2_, although a variety of cost-effective sensor technologies were evaluated, including thin film and MOS, NDIR not only proved to be the most effective but also produced sensors with digital outputs, which can be easily integrated into software. Whereas the NDIR sensors return CO_2_ concentration in Parts Per Million (PPM), the electrochemical sensors respond to the gas being measured by either oxidising or reducing it, generating a very small positive or negative current, i.e., tens of nanoamperes, proportional to the presence of the gas. To accurately measure these reactions, as recommended by the sensor manufacturers, these currents are first converted into voltages using a transimpedance amplifier, and then these voltages are amplified within a range of 0 to 5 V, and finally they are digitised with a 16-bit resolution analogue-to-digital converter. The CO, O_3_, and CO_2_ sensors, along with temperature, humidity, and atmospheric pressure sensors, were mounted on a printed circuit board, designed by Technocomm Consulting Limited. This setup was housed in a plastic box (20 × 9 × 6 cm), creating a compact and cost-effective air pollution monitoring device, EnviroSense™.

To compare their measurements with some ’ground-truth’, these sensors are co-located with those of a reference air quality laboratory, i.e., the Weybourne Atmospheric Observatory (WAO). Established in 1992 and operated by the University of East Anglia, the WAO is a regional station of the Global Atmospheric Watch programme of the World Meteorological Organization. Located on the North Norfolk Coast, UK (52°57′02″ N, 1°07′19″ E, 15 m above sea level), it encounters a broad spectrum of pollution levels, primarily due to southwesterly winds that carry polluted air from various parts of the UK, including London and the Midlands [92]. In particular, since 2008, it has been collecting high-precision long-term in situ measurements of atmospheric carbon dioxide, oxygen, carbon monoxide and molecular hydrogen every minute.

**Figure 1:**
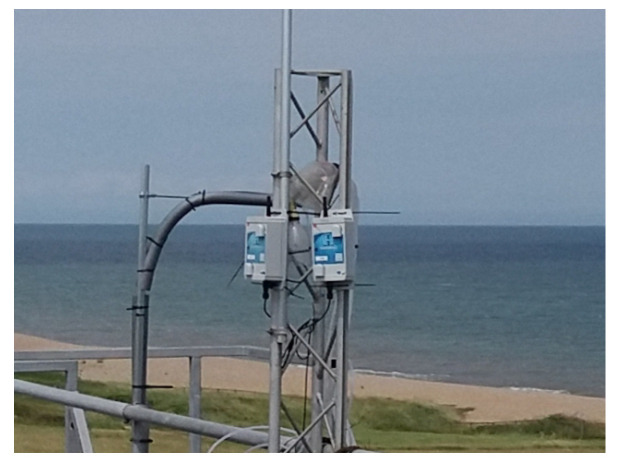
View of the co-located equipment at WAO: cost-effective devices 5158 and 5178 are the two blue and grey boxes.

In order to be able to study sensor consistency, two sets of the cost-effective devices are co-located with this high-precision equipment, see Figure 1. The characteristics of all the gas sensors used in this study are summarised in Table 1.

**Figure 2:**
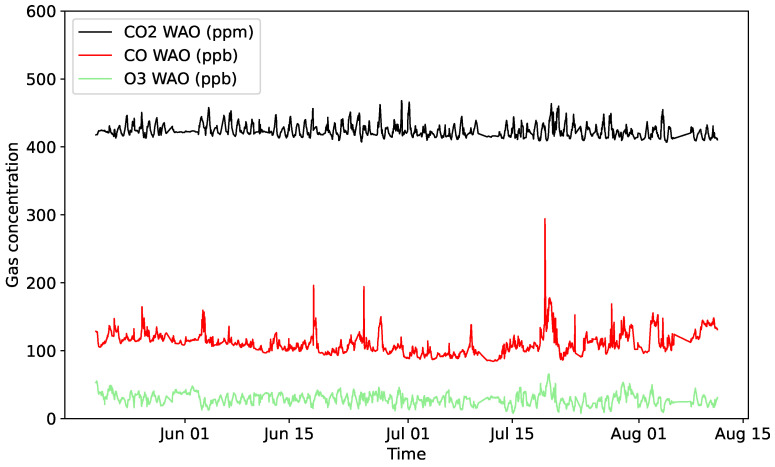
CO, O_3_, and CO_2_ measurements by WAO for the whole period (20 May 2024 to 11 August 2024).

These three sets of data measurements are referred to in this manuscript as from ‘WAO’, ‘5158’, and ‘5178’ (as they are the series numbers of the two cost-effective devices) data. Such data were retrieved continuously for 12 weeks from 20 May 2024 to 11 August 2024. Values were averaged over 30 min for every variable in order to have one observation every 30 min as a compromise between time resolution and measurement noise, leading to up to 4032 observations per individual sensor. Although the data from devices 5158 and 5178 are complete, the gas concentrations measured by the WAO sensors have up to 10% missing values, which was due to various sensor calibration processes having taken place during the period of data collection. Table 2 shows a brief description of the collected dataset, where the minimum, maximum, mean, standard deviation, and number of missing values are provided for each individual sensor. It is important to highlight significant differences in terms of temperature, relative humidity, and pressure measurements between those reported by the WAO and the cost-effective devices (i.e., 5158 and 5178). They arise because the cost-effective sensors collected data within the boxes that house them, while the WAO sensor recorded outdoor measurements. For example, temperature is affected by both the small amount of internal heat produced by the operating electronic components in the box and its direct sunlight exposure. However, the device’s temperature, humidity, and pressure sensors do not aim to measure external conditions; it is by design that they are located inside the box close to the gas sensors to allow for the most effective compensation of their readings. Finally, Figure 2 shows CO, O_3_, and CO_2_ concentrations measured by the reference sensors from the Weybourne Atmospheric Observatory for the 12-week duration of this study.

### 3.2. Sensor Calibration

Calibrating low-cost sensors, especially electrochemical ones, is crucial to ensure accurate measurements. Typically, two-point calibration is used for each sensor: this involves calibrating the sensor at two known concentrations of the target gas, i.e., a zero point using pure air and a span point using a known concentration of the target gas. This process may be repeated a number of times to increase accuracy. As the way this process is performed and the quality of the reference gases affect calibration, in this study, an alternative optimised process is employed: linear regression is used to find the best fit between 11 weeks of data collected by a cost-effective gas sensor and its corresponding WAO sensor. Note that the same 11 weeks are used to train machine-learning-based correcting models, while the remaining week is exploited to assess their performances. As Table 5 shows, correlations between the WAO and calibrated cost-effective sensor readings vary between 72% and 84%, highlighting some limitations of the cost-effective solutions.

**Figure 3:**
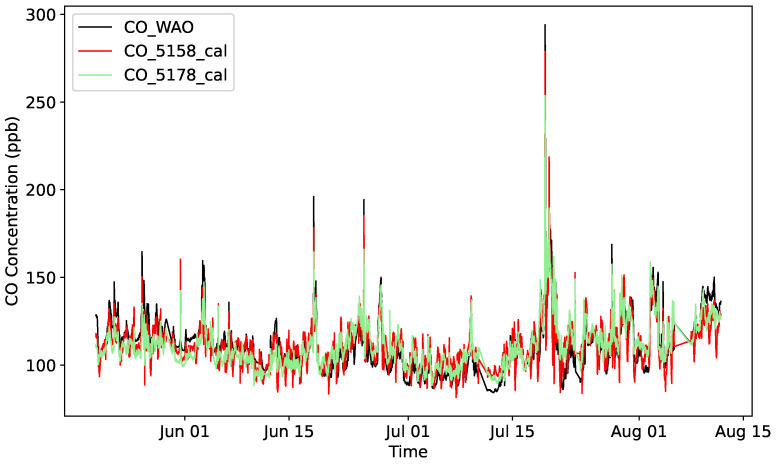
CO measurements after calibration of electrochemical sensors using linear regression.

The outcome of the calibration process for the CO sensors is illustrated in Figure 3. It reveals that the measurements of the cost-effective CO sensors, shown in red and green, generally behave similarly to those of the reference WAO CO sensor. Still, it can be noticed that the readings of the cost-effective sensors are noisier and often do not agree with each other.

### 3.3. Data Analysis

**Figure 4:**
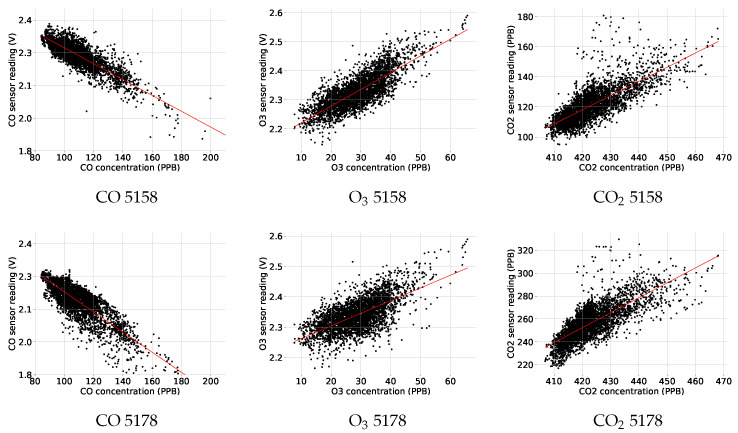
Scatter plots of gas measurements by devices 5158 and 5178 against those by the WAO sensors. Correlations between measurements are illustrated by the red trend lines.

To visualise the relationship between the measurements of the cost-effective gas sensors and those of the reference WAO CO sensor, a scatter plot is produced for each of the gases of interest for both devices, i.e., 5158 and 5178, see Figure 4. For all three gases on each device, a linear relationship is highlighted even if there is some scattering.

**Figure 5:**
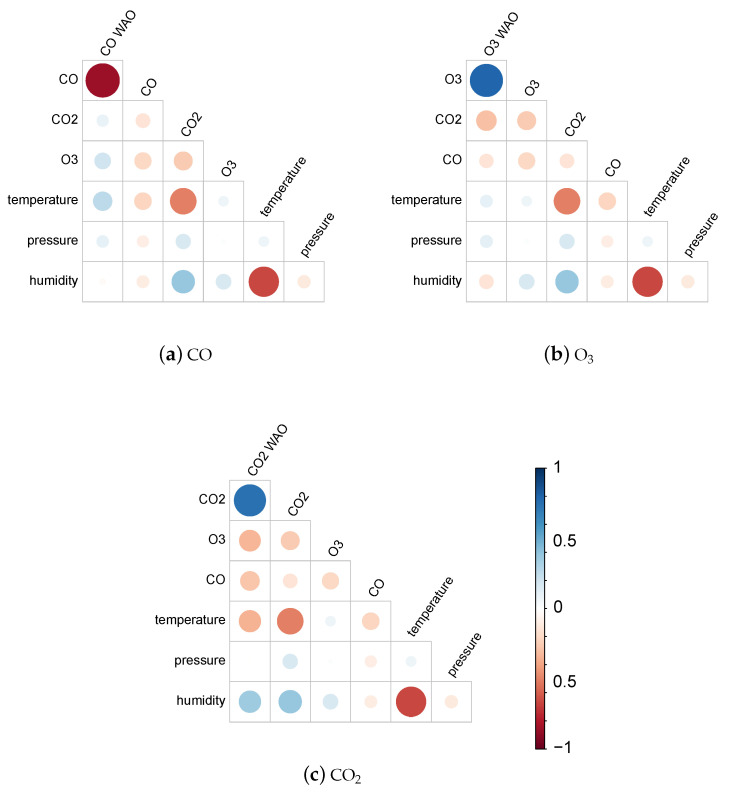
Correlation plots of measurements made by device 5158 with respect to gas readings from the WAO gas sensors.

To investigate this scattering, correlations between gas measurements made by each WAO sensor and all values returned by one of the cost-effective devices are calculated. Figure 5 displays these correlation values for device 5158.

Figure 5a shows that the concentrations of CO measured by the cost-effective device are, as expected, highly correlated to the measurements of CO by the reference sensor. However, there are also important correlations with O_3_ and temperature. This is consistent with the fact that electrochemical sensors are known to display gas cross-sensitivities [25,60,61]. More evidence of gas measurements potentially affected by the presence of other gases and meteorological conditions is provided in Figure 5b,c, where O_3_ seems to be influenced by CO_2_ and CO, and CO_2_ readings are highly correlated to temperature, humidity, and O_3_.

**Figure 6:**
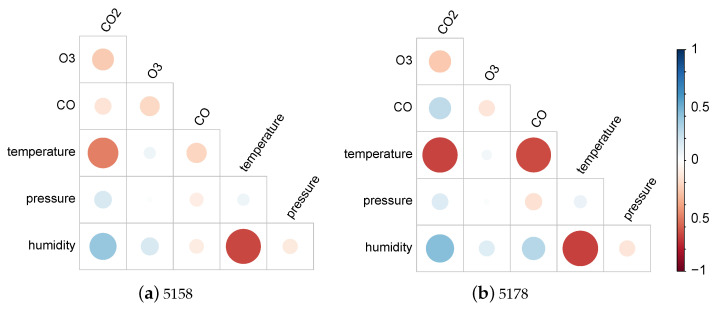
Correlation plots of measurements made by devices 5158 (**a**) and 5178 (**b**).

Finally, Figure 6 highlights slightly different behaviours between sensor 5158 and 5178 towards meteorological variables in particular. Indeed, whereas CO shows a high correlation with temperature for sensor 5178, it is more moderate for sensor 5158.

## 4. Methodology to Enhance Measurement Accuracy

Since the low-cost sensors show accuracy limitations and the previous section suggests that they could be at least partially explained by cross-gas sensitivity and meteorological conditions, it is proposed to design a methodology to learn the relationships between gas and meteorological measurements to correct these sensor readings. As each of the two cost-effective devices host three gas sensors, i.e., CO, O_3_, and CO_2_, one can build a model for either each individual sensor, i.e., 6 models, or each sensor type, i.e., 3 models. Herein, both types of models are investigated following a similar methodology. After introducing the model selected in this study to correct data generated by the low-cost sensors, the process of variable selection is described.

### 4.1. Model Choice

First, as in most practical case studies, it is expected that two-point calibration will be used to calibrate the low-cost sensors and no reference data will be available, meaning that models designed to rely on time series will not be suitable as they increasingly diverge. Second, as the processing power of cost-effective devices is likely to be provided by a microcontroller, model complexity must be relatively low. Third, models that provide a certain level of explainability, as opposed to the so-called ’black box’ models, enable more informed decision making and may reduce scepticism among some audiences. Finally, the linear relationships between measured and ground truth values revealed by Figure 4 suggest that the usage of some linear regression model may be sufficient to enhance performance significantly. For these reasons, it is proposed to train a Multiple Linear Regression model (MLR) to correct the measurements performed by the cost-effective sensors. Moreover, to assess the trade-off made between the model’s complexity and its performance, the results will be benchmarked using support vector regression (SVR). Note that, although experiments were also conducted using Random Forest and long short-term memory models, their performances failed to match those of SVR. Thus, only results obtained using MLR and SVR are reported in this manuscript.

The MLR model for a given gas is defined as the following if one assumes *p* features:

$$Y=\beta_{0}+\beta_{1}x_{1}+\cdots +\beta_{p}x_{p}+\epsilon$$

With the following:

- *Y*: the reference observation;
- $\beta_{0}$: the intercept;
- $\left(\beta_{1},\ldots ,\beta_{p}\right)$: the coefficients of the model for each feature;
- $x_{k}$: the observation for the feature *k*, k=1,…,p;
- ϵ: the residual term.

Whereas, for the sensor-specific models, training and testing only involve data collected by the individual sensor of interest, and for the models designed for a type of sensor, training and testing data combine the calibrated measurements from the two corresponding low-cost sensors from devices 5158 and 5178.

### 4.2. Feature Selection

As each low-cost device retrieves readings from temperature, humidity, pressure, CO, O_3_, and CO_2_ sensors, 6 features are available for each model aiming to enhance gas measurements. As both the number of features and model complexity are quite low, a brute force approach was selected to determine the most suitable feature combination (among $2^{6}-1$, i.e., 63) for each model.

The root mean squared error (RMSE) was chosen as the metric to assess the performance of each generated model:

$$\mathrm{RMSE}=\sqrt{\frac{\sum_{i=1}^{n}{\left(Y_{i}-{\hat{Y}}_{i}\right)}^{2}}{n}}$$

where *n* is the number of observations, $Y_{i}$ is the reference measurement, and ${\hat{Y}}_{i}$ is the enhanced measurement, i.e., ${\hat{Y}}_{i}=Y_{i}-\epsilon_{i}$.

The RMSE was computed using cross-validation (11 weeks for training and 1 week for testing), and the best combination was defined as the one that minimised the RMSE across all folds.

The outcome of this process is shown in Table 3, where selected features are highlighted for the three gas models of device 5158 and 5178 and the three combined gas models. For example, the best feature combination to correct the CO measurements by device 5158 is CO and humidity. One can highlight that apart from the CO_2_ sensors, the two devices need different features for the best models to be delivered. In addition, humidity is a unique feature that enhances the performance of all models. Regarding the three combined models, experiments concluded in the three cases that the best feature combination was the usage of all the features. Note that a similar process was followed to determine the best feature combination for the SVR models.

To identify how important the individual features are to the measurement correction models, each feature was standardised before computing the MLR coefficients of each model:

$$Z=\frac{X-\bar{X}}{\sigma (X)}$$

where

- Z: the standardised value of the feature;
- X: the initial value of the feature;
- $\bar{X}$: the mean value of the feature;
- σ(X): the standard deviation of the feature.

**Figure 7:**
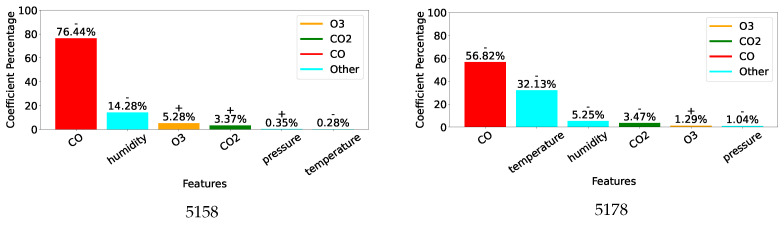
Coefficients for individual CO models.

**Figure 8:**
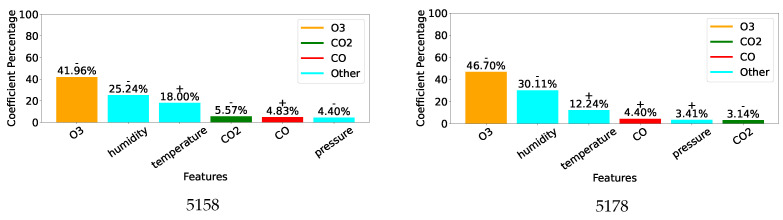
Coefficients for individual O_3_ models.

**Figure 9:**
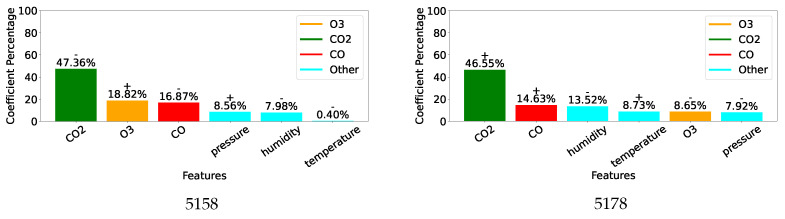
Coefficients for individual CO_2_ models.

Figure 7, Figure 8 and Figure 9 display, for the gas model of each individual sensor, the percentage of the absolute value of the MLR coefficient for each of the 6 features. Overall, as expected, the most important feature for each model is the gas measurement associated with the model with values varying between 42% and 76%. Then, humidity and/or temperature are critical to the CO and O_3_ models, whereas CO and either O_3_ or humidity contribute to more than 13% of the corrections of the CO_2_ models. As already suggested by the different feature correlations displayed in Figure 6, the corresponding gas sensors of the two devices can show significantly different feature contributions.

**Figure 10:**
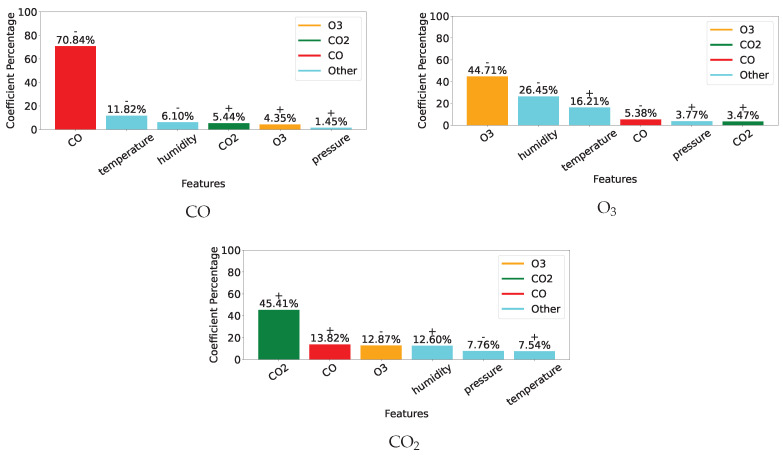
Coefficients for the combined models.

Figure 10 shows similar information for the three combined gas models, where the importance of each feature is consistent with those displayed for the individual models.

## 5. Results

All experiments were conducted using cross-validation with an 11 weeks of training and 1 week of testing dataset. The reported results comprise measurement calibration and enhancement using MLR with both all features and best features (where available) and using SVR with the best features. Note that for each SVR model, the choice of the regularisation parameter (or cost), margin, and kernel (and their associated parameters) was optimised. Using WAO measurements as the ground truth, the performance was evaluated using three main metrics, i.e., Pearson’s correlation coefficient (correlation), the Mean Percentage Error (MPE), and the Mean Average Error (MAE), including the associated standard deviation (STD) and min and max errors.

Two sets of experiments were conducted to evaluate the generated models: first, with models designed for each individual sensor, and second, with combined models associated with individual gases. Note that the corrections delivered by the various MLR models have proved statistically significant as evidenced by the *p*-values obtained from paired *t*-tests, see Table 4.

### 5.1. Models for Individual Sensors

Table 5 reports the results obtained from models tailored to each individual sensor. Correlations between the WAO and calibrated cost-effective sensor readings vary between 72% and 84%, which is usually considered as strong correlations. In addition, there are important correlation differences both between types of gas sensor, with CO sensors performing best, and within a type of gas sensor, especially O_3_.

In terms of enhanced measurements, all models using ’Best features’ outperformed the calibration alone. For example, correlations increased to the range 81% and 91%, and the MAE decreased by up to 46%. This confirms the added value of using additional features to that of the gas of interest. Although feature selection improves results overall, gains are generally quite limited. In addition, SVR models outperformed MLR models offering systematically the best MAE and reduced both the standard deviation and max error.

### 5.2. Models for Combined Sensors

The results obtained by the combined models are displayed in Table 6. In terms of the calibration performance, correlations are around the mean of the values obtained by the two corresponding sensors. As previously, both MLR and SVR enhance measurement quality for O_3_ and CO_2_, with SVR doing better than MLR. However, the CO results are not improved as the various metrics show. This may be explained by the discrepancies between CO sensors 5158 and 5178 as shown in Figure 7. Indeed, it suggests very different behaviours especially towards temperature.

Comparison with the performance reported in Table 5 clearly shows that models targeting single sensors better enhance measurements than the more general ones designed for a sensor type. Still, one can observe that for O_3_ and CO_2_ sensors, the combined models provide some added value when compared to the performance delivered by individual sensor calibration.

## 6. Discussion

This study provided valuable insights into the limitations of three types of low-cost sensors: two electrochemical sensors for CO and O_3_ and a non-dispersive infrared sensor for CO_2_. In addition, it evaluated how the usage of Multiple Linear Regression models can be exploited to correct sensor readings.

Given the 12-week duration of data collection, this work was unable to assess the impact of sensor ageing. However, it provides evidence supporting several other causes of measurement inaccuracies described in Section 2.3. Although sensor manufacturers only provide correction curves for temperature, Figure 5 and Figure 6 reveal significant correlations between individual gas measurements and not only temperature but also humidity and other gases. This is particularly evident for the non-dispersive infrared sensors measuring CO_2_ and the electrochemical sensors reporting CO concentration. Inconsistent behaviour between two units of the same sensor is also noticeable, especially between the two CO sensors, which were affected very differently by temperature and humidity. This is clearly highlighted by the very different coefficient values of their individual models designed to correct their readings, see Figure 7.

For models trained on each individual sensor, the use of MLR models often significantly enhances performance. One should note that feature optimisation has shown very limited impact. Conversely, results using SVR suggest that employing more sophisticated models could further improve measurements if the constraint of processing data on the device was removed. Nonetheless, additional gains might be limited, as previous studies highlight cross-sensitivity with other gases, including Cl_2_, NO_2_, and H_2_S [25,60,61], and the value of integrating meteorological data [25,26,28,88,89]. Thus, a promising strategy to enhance gas readings could involve feeding the models with more relevant features that they could exploit: air pollution monitoring devices could be equipped with additional sensors to detect other types of gases, and/or they could retrieve external meteorological information.

While ideally, a single correction model for each type of gas sensor would be preferred, the performance reported in Table 6 and the cross-sensitivity of individual units highlighted in Figure 7, Figure 8 and Figure 9 indicate that similarity between units’ behaviour is crucial for the success of such models. In this study, the combined model proved very successful for the two O_3_ sensors, which share very similar sensitivity profiles, see Figure 8. However, the model failed to deliver enhanced measurements for the two CO sensors, which exhibit very different sensitivity to other parameters, see Figure 7. Indeed, significant inter-unit inconsistency is frequently observed in electrochemical sensor systems [93]. Given that it may be unrealistic to expect significant improvements in the manufacturing consistency of low-cost sensors in the short term, a practical approach could be to design a small range of models for each sensor type and use factory calibration to associate each unit with one of these models.

## 7. Conclusions

The primary objective of this study was to assess how machine learning algorithms could improve readings from low-cost sensors, ensuring that these corrections could be performed on the monitoring device, i.e., on a simple microcontroller. To meet these requirements, Multiple Linear Regression models were trained and tested using data collected from two electrochemical sensors measuring CO and O_3_ concentrations, as well as a non-dispersive infrared sensor estimating CO_2_ levels. These sensors were co-located with an air quality monitoring reference station to ensure validation against high-precision measurements.

Models designed for a specific sensor unit significantly enhanced measurements, increasing their correlations with those from reference-grade instruments by up to 10%. Further improvements in accuracy are anticipated by incorporating additional features into the models, such as meteorological data and extra gas readings. Naturally, if the correction process could be performed online, more sophisticated machine learning models could be considered. However, this study also revealed that variations in the behaviour of different sensor units can hinder the development of a single correction model for each sensor type. To address production inconsistencies, if a factory-based calibration process was able to classify units according to sub-types, specific correction models could be developed for each of them.

While challenges persist, there is confidence that they will soon be overcome, enabling cost-effective devices to provide dense air quality monitoring networks. Such advancements would contribute significantly to several United Nations Sustainable Development Goals, promoting broader access to reliable environmental monitoring.

## Figures and Tables

**Table 1:** Specifications of the sensors used in this study. Note that PPB and PPM are Parts Per Billion and Million, respectively.

Sensor Type		Weybourne Atmospheric Lab	Cost-Effective Solution
CO	Model	Ecotech Spectronus	Honeywell AQ7CO
	Technology	Fourier Transform Infrared Spectrometer	Electrochemistry
	Precision	1 PPB	Unknown
	Unit cost	>USD 100,000 (it measures both CO and CO_2_)	<USD 150
O_3_	Model	Thermo 49i Ozone Analyser	Honeywell AQ7OZ
	Technology	UV Absorption	Electrochemistry
	Precision	1 PPB	Unknown
	Unit cost	>USD 3000	<USD 150
CO_2_	Model	Ecotech Spectronus	Sensirion SCD30
	Technology	Fourier Transform Infrared Spectrometer	Non-dispersive infrared
	Precision	100 PPB (0.1 PPM)	30,000 PPB (30 PPM)
	Unit cost	>USD 100,000 (it measures both CO and CO_2_)	<USD 50
Others		Temperature (°C), relative humidity (%), and pressure (hPa)	

**Table 2:** Description of the data collected by each sensor.

Sensor	Unit	Min	Max	Mean	Std. Dev.	Missing Values
Temperature WAO	°C	7.56	28.07	14.93	3.20	2
Temperature 5158	°C	13.85	37.74	22.26	3.97	0
Temperature 5178	°C	12.99	38.07	21.69	4.08	0
Relative humidity WAO	%	32.31	100.00	77.29	13.47	2
Relative humidity 5158	%	27.22	68.41	52.03	9.04	0
Relative humidity 5178	%	26.21	70.21	54.03	9.77	0
Pressure WAO	hPa	990.52	1023.53	1008.82	5.99	2
Pressure 5158	kPa	99.22	102.53	101.06	0.60	0
Pressure 5178	kPa	99.23	102.54	101.07	0.60	0
CO WAO	ppb	84.25	294.13	110.80	15.76	318
CO 5158	µV	1,380,450	2,382,109	2,232,868	66,708.54	0
CO 5178	µV	1,177,112	2,280,425	2,129,110	85,833.71	0
O_3_ WAO	ppb	7.72	65.69	28.83	8.34	395
O_3_ 5158	µV	2,197,081	2,552,181	2,343,918	49,237.40	0
O_3_ 5178	µV	2,164,612	2,589,412	2,341,934	52,920.40	0
CO_2_ WAO	ppm	406.88	468.03	423.47	9.22	330
CO_2_ 5158	ppm	94.58	180.73	121.17	11.52	0
CO_2_ 5178	ppm	217.99	329.56	256.31	15.89	0

**Table 3:** Features selected for each model by minimising the RMSE.

	5158			5178			Combo (5158 + 5178)		
	CO	O_3_	CO_2_	CO	O_3_	CO_2_	CO	O_3_	CO_2_
CO	√	√	√	√	.	√	√	√	√
O_3_	.	√	√	√	√	√	√	√	√
CO_2_	.	√	√	.	√	√	√	√	√
Temperature	.	√	.	√	.	.	√	√	√
Humidity	√	√	√	√	√	√	√	√	√
Pressure	.	√	√	.	.	√	√	√	√

**Table 4:** *p*-values from the paired *t*-tests used to assess the statistical significance of the corrected measurements obtained through various MLR models.

	5158	5178	Combo (5158 + 5178)
CO	$3.18\;\times \;e^{-11}$	$4.34\;\times \;e^{-221}$	$1.09\;\times \;e^{-117}$
O_3_	$8.12\;\times \;e^{-120}$	$9.70\;\times \;e^{-47}$	$5.96\;\times \;e^{-290}$
CO_2_	$1.93\;\times \;e^{-65}$	$1.76\;\times \;e^{-79}$	$4.34\;\times \;e^{-268}$

**Table 5:** Evaluation of calibrated and enhanced measurements for individual sensors against ground truth. Best performance is highlighted in bold for each metric in each experiment.

		Correlation	MPE	MAE	STD	Min	Max
CO 5158 (ppb)	MLR (all features)	**0.84**	4.91%	7.03	4.83	**0.03**	29.44
	MLR (best features)	**0.84**	4.66%	6.86	4.76	0.05	30.10
	SVR (best features)	0.83	4.70%	**6.13**	**4.02**	0.06	**24.65**
	Calibration	0.82	**4.51%**	6.93	4.96	0.04	29.90
CO 5178 (ppb)	MLR (all features)	0.90	**2.95%**	4.80	3.76	**0.02**	29.76
	MLR (best features)	0.90	2.97%	4.78	3.68	0.03	28.84
	SVR (best features)	**0.91**	3.07%	**4.17**	**2.89**	0.04	**20.43**
	Calibration	0.84	5.71%	7.66	4.80	0.06	26.87
O_3_ 5158 (ppb)	MLR (all features)	0.89	8.14%	3.24	2.40	0.02	12.81
	MLR (best features)	0.89	8.14%	3.24	2.40	0.02	12.81
	SVR (best features)	**0.91**	**4.45%**	**2.51**	**1.86**	**0.01**	**9.79**
	Calibration	0.80	11.81%	4.18	3.05	**0.01**	14.56
O_3_ 5178 (ppb)	MLR (all features)	0.82	13.44%	4.93	3.15	0.09	18.83
	MLR (best features)	0.82	13.02%	4.75	3.07	0.06	18.40
	SVR (best features)	**0.85**	**8.40%**	**3.54**	**2.48**	**0.03**	**14.14**
	Calibration	0.72	15.10%	5.19	3.70	**0.03**	18.57
CO_2_ 5158 (ppm)	MLR (all features)	0.80	0.33%	4.18	3.57	0.02	18.85
	MLR (best features)	0.81	**0.32%**	4.14	3.56	0.02	18.88
	SVR (best features)	**0.82**	0.38%	**3.93**	**3.24**	0.02	**16.50**
	Calibration	0.74	0.33%	4.70	3.78	**0.01**	20.35
CO_2_ 5178 (ppm)	MLR (all features)	0.85	0.42%	3.84	3.39	0.03	19.35
	MLR (best features)	0.86	0.42%	3.82	3.40	**0.02**	19.11
	SVR (best features)	**0.87**	**0.41%**	**3.56**	**3.13**	0.03	**16.05**
	Calibration	0.79	0.51%	4.60	3.72	**0.02**	19.38

**Table 6:** Evaluation of calibrated and enhanced measurements for combined sensors against ground truth. Best performance is highlighted in bold for each metric in each experiment.

		Correlation	MPE	MAE	STD	Min	Max
CO (ppb)	MLR (all features)	0.81	**4.27%**	**6.83**	4.99	0.03	35.85
	SVR (all features)	0.81	4.48%	7.07	5.34	0.03	43.21
	Calibration	**0.82**	4.57%	6.85	**4.88**	**0.02**	**31.43**
O_3_ (ppb)	MLR (all features)	**0.85**	10.79%	4.05	2.89	**0.01**	18.74
	SVR (all features)	**0.85**	**9.67%**	**3.89**	**2.85**	**0.01**	**18.01**
	Calibration	0.76	12.33%	4.51	3.35	**0.01**	18.39
CO_2_ (ppm)	MLR (all features)	0.79	**0.29 %**	4.19	3.62	**0.01**	**20.38**
	SVR (all features)	**0.80**	0.32%	**4.01**	**3.60**	**0.01**	20.52
	Calibration	0.75	0.32%	4.56	3.73	**0.01**	20.85

## Data Availability

The dataset is available upon request from the authors.
